# The effect of swallowing rehabilitation on quality of life of the dysphagic patients with cortical ischemic stroke

**Published:** 2017-10-07

**Authors:** Kadir Bahcecı, Ebru Umay, Ibrahim Gundogdu, Eda Gurcay, Erhan Ozturk, Sibel Alıcura

**Affiliations:** 1 Physical Medicine and Rehabilitation Clinic, Ankara Diskapi Yildirim Beyazit Training and Research Hospital, Ankara, Turkey; 2 Otolaryngology-Head and Neck Surgery Clinic, Ankara Diskapi Yildirim Beyazit Training and Research Hospital, Ankara, Turkey

**Keywords:** Dysphagia, Stroke, Quality of Life

## Abstract

**Background:** Swallowing and swallowing-related quality of life studies following stroke were almost always performed by including both patients with brainstem and cortical involvement. It was aimed in this study to show the presence of dysphagia in patients with only cortical ischemic stroke and to investigate the interaction between dysphagia and quality of life as well as to evaluate the effect of a rehabilitation program in the acute phase.

**Methods:** Seventy-two patients with cortical stroke (between 0 and 30 days) and dysphagia were included. Swallowing function of patients was assessed by dysphagia screen questionnaire and fiberoptic endoscopic assessment. Also, functional impairment and swallowing quality of life were assessed. The swallowing rehabilitation program for 4 weeks was given to all patients.

**Results:** All patients demonstrated disorders related to oral phase (n = 69, 95.8%), pharyngeal phase (n = 4, 5.6%) or both phases. The swallowing function, swallowing quality of life and functional impairment were improved at the end of therapy.

**Conclusion:** Swallowing quality of life is severely affected in cortical hemispheric stroke patients and can be improved with an early rehabilitation program.

## Introduction

Dysphagia is defined as disturbance of bolus flow from the mouth to esophagus and it is a severe problem in various neurological diseases and is associated with increased morbidity and mortality.^1^

Stroke is the most common neurological cause of dysphagia. Severe dysphagia is usually observed during the first 2-4 weeks, with a prevalence ranging from 29%-81%.^1^^-^^3^ However, minor swallowing disorders have been reported as the rate of 91% in stroke.^4^

Dysphagia may cause to important complications such as hydrational and nutritional deficiency, aspiration pneumonia and even death.^1^^-^^3^ Aspiration pneumonia is seen in patients with dysphagia during the first year, with a mortality rate as up to 45%.^5^ Studies have been reported that if it is early diagnosed and treated, the complications may be reduced or even prevented.^6^ Moreover, it has been reported that when a person has a dysphagia, the ability to enjoy almost all of life is affected. A minor or intermittent dysphagia can lead to stress in both psychological and social situations. Episodes of choking can lead to a fear of eating that can result in malnutrition and social withdrawal.^7^^,^^8^

Since central control mechanism of swallowing is located in the brainstem, severe dysphagia is more likely to occur when stroke involves this part.^9^ However, unilateral cerebral hemispheric infarction is seen more often than brainstem events.^10^ Despite the frequency of dysphagia is typical in the brainstem or bilateral infarct, it is often seen subsequent to one-sided hemispheric infarct in our daily clinical practice. Swallowing studies have been generally concentrated on the brainstem, ischemic and hemorrhagic types of stroke, but patients with brainstem and cortical involvement were evaluated in combination.^11^^,^^12^ In these studies, most of the hemispheric strokes involve middle cerebral artery (MCA) and perfusion areas. Elaborating more on demographic characteristics of these studied populations indicated that severe dysphagia has been observed in these patients. However, studies have not been performed on ischemic stroke patients with only hemispheric cortical involvement and there is also no study investigating the dysphagia-related quality of life among these patients. Therefore, we aimed to reveal dysphagia in cortical stroke and to demonstrate its impact on quality of life.

## Materials and Methods

This study was performed on 72 patients who referred to our Physical Medicine and Rehabilitation (PMR) clinic between 2015 and 2016. 

The subjects, ranged from 50 to 75 years and applied for rehabilitation of some problems such as functional impairments in the first 30 days after ischemic hemispheric MCA stroke, approved by magnetic resonance imaging, and had dysphagia which was shown by dysphagia questionnaire, were included. This dysphagia questionnaire that our standard used in clinic includes a neurologic examination test and a water drinking test as well as oxygen saturation measurement by pulse oximetry.

The neurological examination test included some abilities such as head lifting, independent seating balance and cranial nerves related to deglutition. On the basis of this, neurological measure outcome (NMO) was created. Based on monitoring and documentation of the water drinking test, swallowing outcome (SO) was calculated. Dysphagia outcome (DO) was calculated by combining the scores for NMO and SO. Accordingly, patients with a score of 3 and below were evaluated as normal.

Seventy-two patients were included between 4 to 15 scores according to DO score in this study. Patients with a history of tumor, head-neck operation, past stroke, known swallowing disorders, the presence of gastroesophageal reflux disease, dementia (mini-mental test < 15) or psychiatric diseases, brainstem, hemorrhagic, subcortical and/or bilaterality, and smoking were excluded. In addition, exclusion criteria for flexible fiberoptic endoscopic evaluation of swallowing (FEES) method were the severe infective disease, bleeding risk and/or decompensated cardiac disease.

Patients and their caregivers were given information regarding the study and their inscriptive approvals were obtained before starting the study. The confirmation of the Ethics Committee of the hospital was taken. The study was carried out in conformity with the standards of the Helsinki Declaration.

Characteristics of 72 patients including age, gender, education, dominant side, additive diseases and disorders associated with respiratory and dental, infarct area, and passing time following stroke were documented. Educational status was as illiterate, under 5-year, 5-year, 8-year, 11-year and over 11-year education.

The motor available condition was evaluated by Brunnstrom motor level for upper and lower limbs as well as hand, separately and graded between 1 and 6. 

***The assessment of swallowing function:*** Swallowing disorders were evaluated using dysphagia screen questionnaire and FEES.

***Mann assessment swallowing ability (MASA) test:*** MASA was applied to a screen test. Twenty-four areas were assessed such as vigilance, communication, hearing, speech and respiration disorders, movement limitation, weakness and incoordination in swallowing muscles, the presence of reflexes associated with swallowing. The score was calculated between 38 and 200 points.

***FEES:*** The test was conducted using a 3.4 mm-diameter fiberoptic nasopharyngoscope. Evaluations were applied in a seated situation or a vertical posture to the utmost. Local anesthetics were not applied to prevent its side effects. To evaluate penetration, aspiration and presence of residue, water was used as the fluid, yogurt and a biscuit as the consistency food. The function of the pharyngeal stage was assessed with these foods and findings were saved as the video images. Swallowing status was determined between 1 and 6 according to the endoscopic measurement scale generated by Warnecke, et al.^13^ According to this, 1 point was defined as normal swallowing, while 2-6 points as dysphagia. The swallowing abnormalities (i.e. oral or pharyngeal stage or both) were detected and noted with respect to results of swallowing assessment procedures.

***Other evaluation parameters:*** The functional status was measured with functional independence measure (FIM), which evaluates two important parts of functions as a motor and cognitive status. This scale includes 18 items and 6 parts comprised of personal care, continence, mobility, transfer, interaction and social cognition; and each item has score between 1 and 7. The total score is between 18 and 126.

Swallowing-related quality of life scale (SWAL-QOL) was used to evaluate the impact of swallowing disorders on quality of life. It was developed by McHorney, et al.^14^ to evaluate the quality of life in patients with oropharyngeal dysphagia. SWAL-QOL contains 44 questions on domains of eating disorder, duration of eating, desire to eat, choice of meal, communication, anxiety, mental health, social functioning, fatigue, and sleep. Each question is evaluated by a score ranging between 1 (the worst) and 5 (the best) points. Each domain can be evaluated separately. In our study total scoring was used.

***Study evaluation protocol:*** The study was performed by specialists that composed of swallowing team members in our hospital as blinded to therapy distribution. The dysphagia screen questionnaire, FIM and SWAL-QOL were applied by the 1^st^ PMR expert on the 1^st^ day of hospital admission. Afterwards, patients were evaluated with the endoscopic method by a blinded otolaryngology specialist and were sent to the 2^nd^ PMR specialist.

***Rehabilitation methods:*** Daily care for oral hygiene training and the required swallow maneuvers, head and trunk positioning and diet modification were given according to the condition of swallowing disorder of all patients. Furthermore, oral motor-strengthening exercises for lips, tongue and jaw, cold-tactile stimulation as well as intermittently or alternatively galvanic stimulation to bilateral masseter or submental muscles, according to the presence of an oral or pharyngeal disorder or both, were received by the same physiotherapist. This program was performed for 4 weeks, 20 sessions in total (1 hour a day and 5 hours per week). Apart from these, cognitive, respiratory, sensorial and motor rehabilitation therapies were given to all subjects.

***Comparisons:*** Dysphagia severity level defined by the FEES and MASA, as well as FIM and SWAL-QOL scores were reevaluated after therapy. The results of therapy and changes within the group were compared.

***Statistical analysis:*** SPPS software (version 22, IBM Corporation, Armonk, NY, USA) version for Windows was used for statistical analysis. Shapiro-Wilk test was used to know whether the quantitative data are normally distributed or not. Descriptive statistics were presented as the mean ± standard deviation (SD) or median (minimum-maximum) for quantitative data and frequencies and percentages (%) for qualitative data. Statistically critical differentiations in recurrent evaluations within the group were shown with the Wilcoxon signed-rank test. The Bonferroni correction was performed to avoid potential Type I mistakes in within-group comparison (P < 0.025).

## Results

A total of 72 patients with hemispheric ischemic patients aged between 50-75 years, who were admitted to hospital as an inpatient, treated in our center during the first 30 days following stroke and were enclosed in the study.

The mean DO which defined the patients as dysphagia was 9.32 ± 2.45. At hospital admission, 8 patients (11.1%) received regimen-3 (normal) diet comprising liquid, semi-solid, and solid foods, 42 patients (58.3%) received regimen-2 diet consisting of semi-solid foods supplemented with intravenous infusion of fluids, 18 patients (25.0%) were fed with nasogastric catheter (n = 18, 25%), and 4 patients (5.6%) received gastrostomy catheter. 

The mean age of the 72 patients was 63.32 ± 11.17 years. Among 72 patients, 25 (34.7%) were female, while 47 (65.3%) were male. The patients had right (n = 61, 84.7%), and left (n = 11, 15.3%) hand dominance. In all patients, ischemic stroke involved MCA region (100%). The mean passing time after stroke was 16.51 ± 8.32 days. Demographic and disease characteristics of subjects are presented in table 1.

According to Brunnstrom staging of the motor functions of the patients at admission, median motor function stage of the upper and lower extremities, and hands of the patients were 2.00 (2.19 ± 1.34), 2.00 (2.53 ± 1.42), and 1.00 (2.12 ± 1.09), respectively.

**Table 1:** Demographic and disease characteristics of subjects

**Feature**		**Value**
Age (year) (mean ± SD)		63.32 ± 11.17
Sex [n (%)]	Female	25 (34.7)
	Male	47 (65.3)
Passing time after stroke (day) (mean ± SD)		16.51 ± 8.32
Educational status [n (%)]	Illiterate	8 (11.1)
	Under 5-year	3 (4.2)
	5-year	48 (66.7)
	8-year	8 (11.1)
	11-year	3 (4.2)
	Over 11-year	2 (2.7)
Dominant side [n (%)]	Right	61 (84.7)
	Left	11 (15.3)
Additive diseases [n (%)]	Hypertension	61 (84.7)
	Coronary artery disease	22 (30.6)
	Diabetes mellitus	11 (15.3)
	Respiratory disease	8 (11.1)
	Hyperlipidemia	29 (40.3)
Additive problem [n (%)]	Dental problems (loss, poor hygiene)	69 (95.8)

SD: Standard deviation

Distribution of bedside screening test and FEES levels results which demonstrated pre-treatment swallowing functions, functional disability and SWAL-QOL scores are shown in table 2. According to bedside screening test and FEES results, the patients demonstrated disorders related to oral phase (n = 69, 95.8%), pharyngeal phase (n = 4, 5.6%) or both phases (n = 21, 30.6%). 

**Table 2:** The distribution of pre-treatment evaluation parameters

**Evaluation parameter (score range)**	**Mean ± SD**
MASA (38-200)	118.47 ± 28.31
FEES	
Dysphagia stage (1-6)	3.52 ± 1.65
FIM	
Motor score (13-91)	25.13 ± 9.23
Cognitive score (5-35)	17.46 ± 6.23
Total score (18-126)	42.62 ± 6.57
SWAL-QOL score (44-220)	117.63 ± 26.37

SD: Standard deviation; MASA: Mann assessment swallowing ability; FEES: Flexible fiberoptic endoscopic evaluation; FIM: Functional independence measure; SWAL-QOL: Swallowing quality of life

Distribution of bedside screening test and FEES levels results which demonstrated post-treatment swallowing functions, functional disability and SWAL-QOL scores are shown in table 3. At the end of the treatment nutritional requirement of the patients were met with regimen-3 (n = 65, 90.3%) and regimen-2 (n = 6, 8.3%). One patient (1.4%) was persistently fed via gastrostomy catheter. 

**Table 3:** The distribution of post-treatment evaluation parameters

**Evaluation parameter (score range)**		**Mean ± SD**
MASA (38-200)	168.42 ± 21.65	
FEES		
Dysphagia stage (1-6)	1.48 ± 0.92	
FIM		
Motor score (13-91)	29.42 ± 12.45	
Cognitive score (5-35)	28.14 ± 6.87	
Total score (18-126)	57.73 ± 11.18	
SWAL-QOL score (44-220)	151.63 ± 28.21	

SD: Standard deviation; MASA: Mann assessment swallowing ability; FEES: Flexible fiberoptic endoscopic evaluation; FIM: Functional independence measure; SWAL-QOL: Swallowing quality of life

The significant improvement was detected after treatment swallowing functions of the patients (MASA: P = 0.003, and FEES P = 0.004). A significant improvement was detected in cognitive, and total disability scores of functional disability scale using FIM (P = 0.011, and P = 0.023, respectively), while the change in motor function scores was not significant (P = 0.467). Also, a significant improvement was found in SWAL-QOL score (P = 0.001).

## Discussion

Dysphagia in patients with stroke commonly occurs after ischemia of the cerebral cortex. In recent studies, it has been reported that most of these patients had regained their swallowing functions, while in 11-15% of them dysphagia which led to complications as aspiration pneumonia had persisted.^9^^-^^11^^,^^15^^-^^17^ In previous studies, it has been shown that brainstem stroke is a primary risk factor for persistent dysphagia. Because, the swallow response is generated in the brainstem swallowing center located in the medulla oblongata which combined knowledge directed from the oral, pharyngeal and suprabulbar areas. These centers were considered to be autonomous central pattern generators largely controlling the synchronization and timing of swallowing. However, a complex array of cortical representation, including motor, premotor, and sensorimotor cortices, appears to be crucial in its effective coordination.^18^ These cortical areas provide volitional deglutition and supply primarily to trigger swallowing and control the of swallow motor response.^9^^,^^18^^,^^19^ Normal swallowing is generally divided into four stages. However, this division is not as simple as it is said to be. The normal swallow is a complex, fast, continuous sequence of coordinated muscle movements and there is some overlap between the phases. Cortical stroke has been shown to have impact on the pharyngeal phase of the swallow, with impairment to initiation and duration and increased frequency of penetration and aspiration as well as with impairment in pharyngeal transit and longer oral transit.^20^^,^^21^

Brainstem strokes may account for up to 15% of all strokes. In two recently performed studies, in which cortical hemispheric involvement was reported in 90% of the patients who were diagnosed, and followed up with dysphagia at early stroke period, spontaneous return of swallowing functions was indicated in only 9.5% and 37% of the patients, 1 month later in the first, and 3 months later in the second study.^6^^,^^22^ Moreover, in patients with severe dysphagia who require feeding via a gastrostomy tube, no difference has not been detected between brainstem involvements and cortical hemispheric strokes.^23^^,^^24^ The other two studies have been demonstrated that the presence of dysphagia is related to MCA involvement in patients with cortical stroke.^25^

Because of these reasons, in our study, we included 72 patients with ischemic stroke involving perfusion area of MCA. We evaluated our patients for a mean period of 16.51 ± 8.32 days after stroke, which was somewhat longer than conventionally reported recovery time, and detected disorders related to oral (n = 69, 95.8%), pharyngeal (n = 3, 4.2%), and oropharyngeal (n = 21, 30.6%) phases. These patients (n = 72) also represented the whole spectrum of mild to very severe dysphagia. Patients’ SWAL-QOL scores were nearly half of maximum well-being index scores, and general functional impairment levels were one-third of normal scores. We applied a combination treatment also including electrical stimulation on our patients for 4 weeks. 

Generally, spontaneous recovery of swallowing function occurs within the first 2-4 weeks, so in previous studies initiation of a rehabilitation program for dysphagia was postponed after that period. In guidelines for management of dysphagia, treatment of dysphagia is absolutely advised,^26^ while in some studies it was advocated that it would provide beneficial effects if applied at an early stage.^6^ However according to the guidelines on rehabilitation of stroke lack of adequate data have been stated.^27^

In recent years, the presence of a cortical inhibition in both intact and damaged hemisphere, and functional recovery induced by compensatory cortical re-organization have been indicated.^3^^,^^10^ Especially early phase was reported as a window of opportunity.^6^^,^^28^ In a study, in patients who received classical treatment at an early period in stroke within the first 2 weeks 100% improvement was achieved in oral phase problems, and 75-90% recovery in pharyngeal phase disorders with a lesser number of treatment sessions than in patients that same treatment initiated in one month later.

Also, in patients applied treatment after 4 weeks, oral and pharyngeal phase problems were detected which were regressed in 15%, and 45% of the cases, respectively. Aspiration detected video fluoroscopically was persisted in 60% of these patients.^6^

In the light of this information, we also applied combined rehabilitation program for our patients at a considerably early stroke period. Indeed, in studies performed using both traditional methods, and new techniques which involve electrical stimulations, it has been reported that especially combination treatments decreased dysphagic complications and increased rate of oral feeding.^29^^-^^32^ Similar to our study, Bulow, et al. reported that dysphagia treatment was effective even in their subacute phase of patients with hemispheric stroke.^32^

The medical complications of dysphagia include aspiration pneumonia and malnutrition. Other complications of dysphagia in stroke patients are psychological and social effects because eating is an enjoyable social activity, and inability to eat usually may affect patient morale and quality-of-life.^33^^,^^34^

Swallowing-related quality of life has been evaluated using different scales, and different studies have reported that fear from choking during eating, and inability to control dysphagic symptoms, physical, and social insecurity secondary to anxiety and fear are the most frequently encountered problems. In meta-analyses performed, especially evaluation of the quality of life of dysphagic patients has been indicated.^33^^,^^34^ These meta-analyses have been reported that fears of these patients, and their reflections on the social environment to be adverse parameters affecting their quality of life. Since they are most frequently seen especially during the acute phase, quality of life is most affected during this early period. However, these studies have been most frequently performed in cases with brainstem strokes.^16^^,^^34^ We considered that hemispheric strokes are more frequent, and low quality of life may be more prevalent in these patients. Therefore, in our study SWAL-QOL scale was used. The items on the SWAL-QOL address desire for eating, dysphagia symptom frequency, mental health, social concerns related to swallowing problems, food selection, fear related to eating, and the burden of dysphagia. While the quality of life of nearly 50 of our studied patients was deteriorated before treatment, this rate dropped down to 30% after treatment.

Despite lack of similar studies in the literature, our result suggests that dysphagia related to hemispheric strokes is as important as those associated with brainstem strokes with respect to quality of life.

## Conclusion

As a result, cortical strokes are frequently encountered in our clinical practice, and contrary to our classical information they can induce dysphagia which will be able to affect the quality of life. We think that rehabilitation programs that applied these patients in early stroke period will decrease both medical and psychosocial complication rates related to dysphagia.

## Notes:

**How to cite this article:** Bahcecı K, Umay E, Gundogdu I, Gurcay E, Ozturk E, Alıcura S. The effect of swallowing rehabilitation on quality of life of the dysphagic patients with cortical ischemic stroke. Iran J Neurol 2017; 16(4): 178-84.
